# D_3_‐Creatine dilution and the importance of accuracy in the assessment of skeletal muscle mass

**DOI:** 10.1002/jcsm.12390

**Published:** 2019-03-21

**Authors:** William J. Evans, Marc Hellerstein, Eric Orwoll, Steve Cummings, Peggy M. Cawthon

**Affiliations:** ^1^ Department of Nutritional Sciences and Toxicology University of California Berkeley USA; ^2^ California Pacific Medical Center Research Institute, University of San Francisco USA; ^3^ Department of Epidemiology and Biostatistics University of California San Francisco CA USA; ^4^ Department of Medicine Oregon Health and Science University Portland USA

**Keywords:** Sarcopenia, D_3_‐Creatine dilution, Body composition, Skeletal muscle, Duel X‐ray absorptiometry

## Abstract

Sarcopenia has been described as the age‐associated decrease in skeletal muscle mass. However, virtually every study of sarcopenia has measured lean body mass (LBM) or fat free mass (FFM) rather than muscle mass, specifically. In a number of published sarcopenia studies, LBM or FFM is referred to as muscle mass, leading to an incorrect assumption that measuring LBM or FFM is an accurate measure of muscle mass. As a result, the data on the effects of changes in LBM or FFM in older populations on outcomes such as functional capacity, disability, and risk of injurious falls have been inconsistent resulting in the conclusion that muscle mass is only weakly related to these outcomes. We review and describe the assumptions for the most commonly used measurements of body composition. Dual‐energy X‐ray absorptiometry (DXA) has become an increasingly common tool for the assessment of LBM or FFM and appendicular lean mass as a surrogate, but inaccurate, measurement of muscle mass. Other previously used methods (total body water, bioelectric impedance, and imaging) also have significant limitations. D_3_‐Creatine (D_3_‐Cr) dilution provides a direct and accurate measurement of creatine pool size and skeletal muscle mass. In a recent study in older men (MrOS cohort), D_3_‐Cr muscle mass was associated with functional capacity and risk of injurious falls and disability, while assessments of LBM or appendicular lean mass by DXA were only weakly or not associated with these outcomes. Inaccurate measurements of muscle mass by DXA and other methods have led to inconsistent results and potentially erroneous conclusions about the importance of skeletal muscle mass in health and disease. The assessment of skeletal muscle mass using the D_3_‐Cr dilution method in prospective cohort studies may reveal sarcopenia as a powerful risk factor for late life disability and chronic disease.

## Introduction

The assessment of body composition and its relation to health outcomes has been an integral component of medical research. While body mass index (BMI) is used as an index of overweight and obesity, the effects of the major individual components of body mass, including fat and skeletal muscle, on outcomes depends upon the accuracy of the method being used. Body composition may be measured using a number of different methods, each of which rely on assumptions that may not be true in all populations, ages, or clinical conditions.

In particular, the assessment of total body skeletal muscle mass has, until recently, been problematic. Skeletal muscle is a significant but not the only component of lean body mass (LBM) or fat free mass (FFM). In a large number of clinical trials,^1^, ^2^, ^3^, ^4^, ^5^, ^6^, ^7^, ^8^, ^9^ lean mass (or FFM) is incorrectly referred to as muscle mass. We will discuss how the use of FFM as a surrogate for muscle mass has resulted in erroneous conclusions on the importance of skeletal muscle in development of late‐life dysfunction and risk of chronic disease. In this review, we, briefly, comment on a number of commonly used methods for assessing FFM highlighting the assumptions, advantages, and limitations of each. The intent is not to provide a comprehensive review (see Heymsfield *et al*.^10^) of body composition methodology but rather provide a discussion of the assumptions employed with regard to the assessment of whole‐body skeletal muscle mass.

## Lean body mass (also referred to as fat free mass)

### Body density (underwater weighing or whole body plethysmography)

Body density may be accurately assessed using underwater weighing, or more recently, whole body plethysmography to determine fat and FFM.^11^ However, this two compartment model incorrectly assumes that the density of fat and lean tissue is constant in all populations. Because the density of LBM is so highly variable among different ethnic groups, athletic populations, and different ages,^12^, ^13^ LBM assessment using body density is not a valid or reliable estimate of muscle mass.

### Total body water (TBW)

Total body water (TBW) may be accurately measured by delivering a known amount of ^3^H_2_O or ^2^H_2_O and sampling body water (saliva, urine, or blood) after the dose has been completely mixed. TBW has been used as an indicator of FFM and, indirectly, of skeletal muscle mass. This estimate of FFM assumes a constant state of hydration and that skeletal muscle is a constant percentage of FFM. However, hydration status can be highly variable during illness, aging, exercise, and exposure to heat.^14^ Extracellular water volume is highly variable in many common disease states, including heart failure, kidney disease, or oedematous conditions. Chronic exposure to heat and regular exercise in a hot environment can result in a large expansion of plasma volume (up to 20%).^15^ These factors make TBW estimates of lean less accurate and the relative content of muscle mass or lean mass varies by age, conditioning, and clinical status.

### Bioelectric impedance (BIA)

Bioelectric impedance (BIA) measures body conductivity or resistance to a small electrical current through the body or across a limb. The resistance is strongly related to TBW (electrolyte‐rich fluids) and is used to assess LBM and, by subtraction, total fat content.^16^, ^17^ Because BIA provides an inexpensive and easy to use measurement, it has been used in a number of large cohort studies examining the effect of lean mass on a number of outcomes. Janssen *et al*.^18^ used BIA data collected on 4504 men and women over the age of 60 years in the Third National Health and Nutrition Examination Survey to estimate the prevalence of sarcopenia. While BIA does not directly measure skeletal muscle mass, this study demonstrated that low lean mass was independently associated with reduced functional capacity, particularly in older women. A recent study^19^ compared BIA and dual‐energy X‐ray absorptiometry (DXA) estimates of lean mass in 653 men and 3002 women with a range in BMI 16–40. With a *r* = 0.897 (*P* < 0.0001), the investigators concluded that these two methods are interchangeable for estimates of LBM at the population level; however, concordance between the two methods at the individual level is lacking, irrespective of BMI, and the same limitations for assessing muscle mass apply to TBW measured by BIA as for its measurement by isotope dilution (*Table* 1).

**Table 1:** Most commonly used total body composition methods

Method	Basis	Assumptions	Body composition measured
Whole body plethysmography (Bod Pod)^11^	Body volume and weight to determine density	Density of fat and lean mass is constant with age, BMI, and ethnicity	Two compartments, fat and fat free mass
DXA^22^	Absorption of two different energy X‐rays	X‐ray absorption is directly related to bone, fat, and lean mass	Direct measure of bone density, fat, and lean mass does not measure muscle mass
Bioelectrical impedance/bioelectrical spectroscopy^16^, ^17^	Resistance and reactance to a single or multiple frequency (BIS) electrical current	Resistance and reactance values are associated with body water content	Value is strongly associated with total body water, including intracellular and extracellular; does not measure muscle mass
24‐h urine creatinine^34^	Excretion of creatinine in a 24‐h urine collection	No urine is lost during collection period; no dietary creatinine; creatinine excretion reflects total body creatine pool; creatine pool size is a measurement of muscle mass	Total body creatine pool size, skeletal muscle mass
D_3_‐Creatine dilution^37^	Enrichment of D_3_‐creatinine in single urine sample	Urine D_3_‐creatinine represents muscle D_3_‐creatine enrichment; creatine pool size is a measurement of muscle mass	Total body creatine pool size, skeletal muscle mass

### Computerize tomography (CT) and magnetic resonance imaging (MRI)

**Figure 1:**
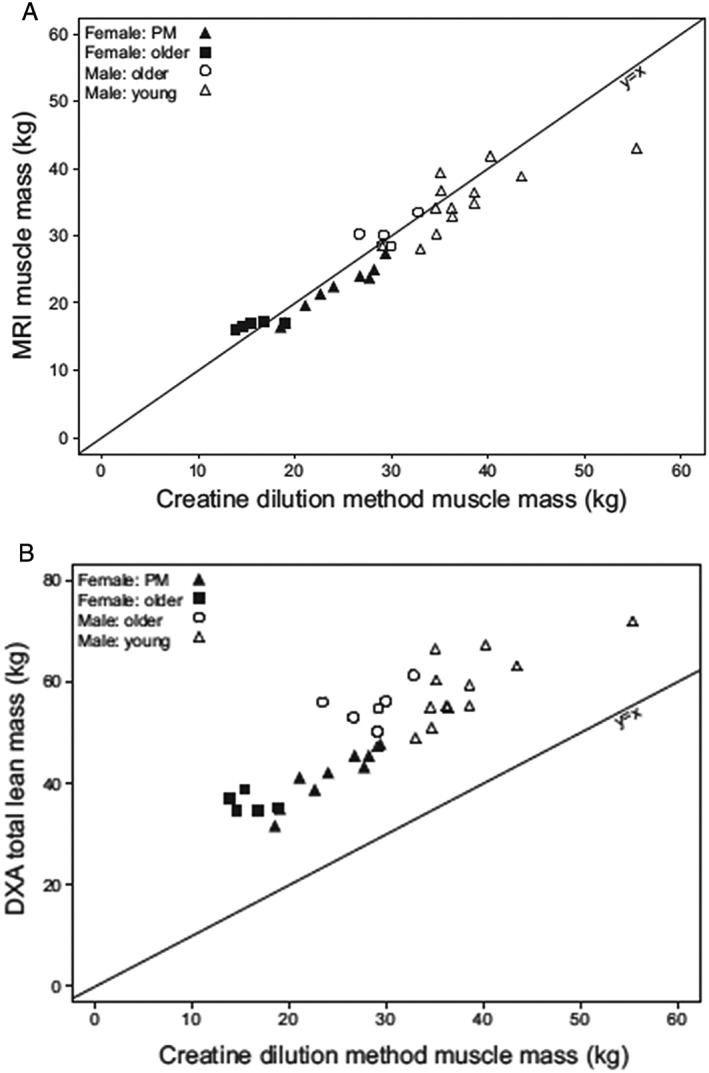
(A) The relationship between whole body MRI vs. muscle mass by D_3_‐creatine dilution (*r* = 0.868, *P* < 0.0001) and (B) between whole body DXA and D_3_‐creatine dilution (*r* = 0.745, *P* < 0.0001).^37^ The correlation in (A) passed through the origin, while in (B), DXA estimates of lean body mass was systematically greater than that of skeletal muscle mass by D_3_‐creatine dilution. DXA, dual X‐ray absorptiometry; MRI, magnetic resonance imaging.

While CT and MRI have been used in clinical trials, each has limitations that reduce applicability for large cohort studies. CT and MRI provide a cross‐sectional measurement of anatomic composition and use of multiple CT or MRI slices can provide estimates of volume of muscle or fat tissues.^20^ CT has also been useful in estimating the lipid content of specific cross‐sectional scans. The National Institute on Aging supported health and body composition study showed that the attenuation score of CT scans is associate with lipid content of the thigh muscles of older men and women^21^ and that increasing age and/or BMI was associated with increased muscle lipid. This increased muscle lipid content was associated with decreased force production and may be a good measure of muscle quality. The assessment of lipid is based on an attenuation score of Hounsfield units which measures the quality of the muscle imaged. CT or MRI measurements of cross‐sectional area or whole body skeletal muscle mass are precise and applicable in small research studies, but the very high cost, limited availability for field studies and radiation exposure (for CT) make these impractical for large scale studies, field research, or routine medical management (*Figure* 1).

### Dual‐energy X‐ray absorptiometry lean mass

**Figure 2:**
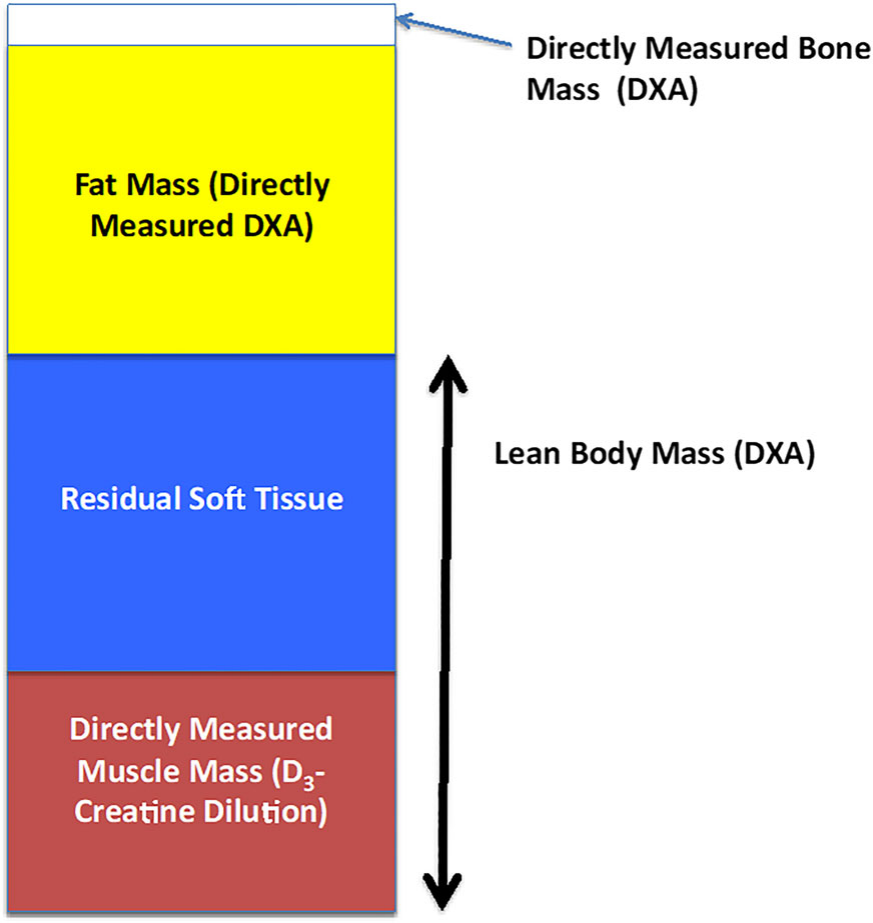
Representative chart of components of body composition measured by dual X‐ray absorptiometry (DXA) and D_3_‐creatine dilution. DXA provides a direct measure of bone and fat mass, but not muscle mass. In subjects where both DXA estimates of lean mass and muscle mass by D_3_‐Cr dilution. While each component of body composition varies by age, sex, and medical condition, muscle mass is about 50% of lean mass. The non‐muscle components of lean mass is termed residual lean mass.^37^, ^42^

The most widely used method in current use to estimate lean mass is DXA. DXA machines are commonly used to measure bone mineral density for the diagnosis of osteoporosis or osteopenia and thus have the potential for widespread clinical use for other purposes, including body composition measures. DXA estimates body composition using a three compartment model; fat and bone mineral content are directly measured through the differential absorption of two photon energies, and then by subtraction, lean mass is determined.^22^ Soft‐tissue lean mass includes muscle mass, water, organ weight, and all other non‐bone and non‐fat soft tissue (*Figure* 2). Therefore, DXA does not measure muscle mass specifically. Baumgartner *et al*.^4^ were the first to use DXA‐based measures of lean mass to operationalize a definition of sarcopenia, which demonstrated that low lean mass of the arms and legs (appendicular lean mass) was associated with worse functional status (although imputed DXA data were used for some participants). Since then, a number of studies have examined the association between DXA lean mass and functional status in older adults, although with mixed results. A meta‐analysis by Schaap *et al*.^23^ found that various measures of muscle size (both lean mass and muscle cross‐sectional area by CT and BIA) were not associated with functional decline, while poor muscle strength was associated with decline. An informal meta‐analysis by Manini and Clark^24^ found, at best, a weak relation between lean mass by DXA and poor performance or function (which did not reach statistical significance). Accordingly, DXA based measures of lean mass do not represent muscle mass and may not be the most appropriate tool to assess risk of functional decline in older persons.

The use of many of these methods for whole body composition of lean and fat mass has provided essential data on effects of diet, disease, exercise, and aging, particularly with regard to total body fat mass and bone density. However, the measurement of one of the principal tissues of body composition, skeletal muscle mass, using these tools is not accurate, and has likely resulted in an erroneous estimate of the effects of muscle mass on outcomes such as risk of disability, falls, and all‐cause mortality.

Nevertheless, a recent review^25^ suggested that DXA should be the reference standard for the assessment of what is termed ‘lean muscle mass’ for future studies examining the role of muscle mass in health and disease. The authors state that ‘based on the feasibility, accuracy, safety, and low cost, dual energy X‐ray absorptiometry can be considered as the reference standard for measuring muscle mass’. The authors also state that different methods of assessment will result in ‘different prevalence of sarcopenia and may therefore have significant consequences on preventive or therapeutic strategies’. This last point raises a critical issue. Although the use of DXA to assess muscle mass is precise, and its feasibility and safety are well known, DXA is inaccurate for measuring muscle mass. As noted earlier, DXA can not in theory and does not in practice provide an accurate measurement of skeletal muscle mass.

A meta‐analysis^23^ of longitudinal observation studies in older people, conducted between 1976 and 2012 (≥65 years) examined body composition (BIA, DXA, and CT) and physical functional capacity. In the studies that examined lean mass (termed muscle mass), the authors concluded that ‘low muscle mass was not significantly associated with functional decline’. They also concluded that the role of muscle mass in the development of functional decline was unclear but was ‘much smaller than the role of fat mass and muscle strength’. These conclusions that the loss of skeletal muscle mass, per se, is only weakly associated with functional outcomes in older people is very likely a result of using the measurement of lean mass as a proxy for muscle mass rather than using a direct measurement of muscle mass.

Indeed, this lack of a strong association between lean mass (as a surrogate measurement of muscle mass) and outcomes in elderly people has led to several consensus definitions of sarcopenia that include measures of muscle strength and/or physical performance in addition to measures of lean mass alone.^26^ A project funded by the Foundation for the National Institutes of Health (FNIH) Biomarkers Consortium Sarcopenia undertook a data‐analysis based effort to better define diagnostic criteria for sarcopenia.^27^ Using DXA derived appendicular lean mass from multiple cohort studies in older people, Cawthon *et al*.^28^ described specific cut points in lean mass that were associated with muscle weakness (grip strength). While the results showed that specific levels of low lean mass were associated with weakness, the association of low lean mass with slow walking speed was inconsistent. Data from the Health ABC study showed that strength (grip or quadriceps) but not lean mass (assessed by CT cross‐sectional area or DXA) was associated with mortality.^29^ Although muscle mass was not measured in this study (only lean mass and cross‐sectional area were assessed), the authors concluded that ‘Low muscle mass did not explain the strong association of strength with mortality, demonstrating that muscle strength as a marker of muscle quality is more important than quantity in estimating mortality risk’. Because of the weak association between lean mass and outcomes, alternative definitions of diagnostic criteria for sarcopenia have incorporated various measures of strength or function.^30^, ^31^, ^32^, ^33^ The consensus definition from FNIH sarcopenia project^27^ cut points for weakness are grip strength <26 kg for men and <16 kg for women, and for low lean mass, appendicular lean mass adjusted for BMI <0.789 for men and <0.512 for women.

### 24‐h Creatinine excretion

The use of creatinine (Crn) excretion provides a direct assessment of whole body creatine pool size and, as a result, of muscle mass. Heymsfield *et al*.^34^ described the measurement of whole body muscle mass whereby the collection of all urine produced during a 24‐h period is used to estimate the total body creatine pool size. This method uses a two main assumptions; (i) almost all of the total body creatine pool is sequestered in skeletal muscle cells and (ii) the conversion of creatine to Crn occurs at a steady and predictable rate (it is well established that Crn is not stored in the body or used and is rapidly excreted). These two assumptions have allowed the excretion of Crn during a specific time period (24 h) to be used to estimate the creatine pool size and, as a result, skeletal muscle mass. This method has been used by a large number of studies as an estimate of muscle mass. Notably, 24‐h urinary Crn excretion was used by investigators at the Baltimore Longitudinal Study on Aging to demonstrate an age‐associated decrease in muscle mass that was associated with declining basal metabolic rate.^35^ They concluded that the decline in basal metabolic rate with aging was strongly associated with declining muscle mass rather than other components of LBM. This method requires collection of all urine produced in a 24‐h period; however, loss of even a single micturition episode in the course of a day, imperfect timing of collections or incomplete emptying of the bladder in the final (usually early morning) sample will decrease precision and decrease the estimate of muscle mass. In addition, diet must be controlled, as the consumption of food containing creatinine (such as meat) will increase 24‐h Crn excretion and lead to an overestimate of total body muscle mass. For these reasons, this method may not be practical for studies with large numbers of subjects.

### D_3_‐Creatine dilution

The D_3_‐creatine (D_3_‐Cr) dilution method uses similar assumptions as the 24‐h Crn excretion assessment of muscle mass: specifically, that about 98% of the total body creatine pool is sequestered in skeletal muscle and that creatine is turned over in muscle through the conversion of creatine to creatinine. The additional assumption of this method is that an oral dose of a tracer quantity of deuterated creatine (D_3_‐Cr) is 100% bioavailable and once absorbed is transported across the sarcolemma and sequestered in sarcomeres. In some subjects, a small amount of the oral tracer dose is ‘spilled’ into urine and not transported into muscle. However, the amount of spillage can be estimated and corrected with an algorithm based on urine levels of creatine and creatinine.^36^ The excretion of Crn and measurement of D_3_‐enrichment in urine Crn provides an opportunity to ‘sample’ the intra‐myocellular enrichment of D_3_‐Cr and thus determines the dilution of the oral label in the whole body creatine pool in skeletal muscle. Importantly, the measurement does not require dietary control and relies on a single spot, fasted urine sample taken 48–96 h after dosing. The enrichment of urine D_3_‐Crn reaches isotopic steady state at about 48 h after dosing and remains stable for about 48 h.^37^ Intramyocellular Cr is turned over at the rate of ~1.7%/day. Because of this relatively slow turnover, subsequent, longitudinal measurements require a pre‐dose urine sample as well as a post‐dose sample, to correct for residual D_3_‐Cr. Cross‐sectional^38^ and longitudinal^39^ validation studies of this method were performed in rats and in humans. The cross‐sectional study in rats of different ages demonstrated a strong relationship between D_3_‐Cr muscle mass and lean mass measured by quantitative magnetic resonance (*r* = 0.959, *P* < 0.0001) and muscle mass of the lower limbs (weighed after dissection) (*r* = 0.929, *P* < 0.0001). A longitudinal validation study of muscle mass accrual of growing animals and loss of muscle mass due to dexamethasone treatment demonstrated a strong relationship between change in quantitative magnetic resonance lean mass and change in D_3_‐Cr muscle mass (*r* = 0.9629, *P* < 0.0001).

A cross‐sectional clinical validation study was performed in young and older men and women.^37^ D_3_‐Cr muscle mass was strongly associated with whole‐body MRI of muscle mass (*r* = 0.868, *P* < 0.0001) and DXA (*r* = 0.745, *P* < 0.0001). The line of identity passed through the origin for whole body MRI vs. D_3_‐Cr muscle while whole body DXA overestimated muscle mass compared with MRI (*Figure* 1). Importantly, the D_3_‐Cr dilution method measures a parameter, the creatine pool, that is associated with skeletal muscle function. In the sarcomere, creatine and creatine phosphate are located adjacent to the Z‐disc and the A‐band^40^ and are not associated with non‐contractile components. This strongly suggests that assessment of the creatine pool size provides an indicator of functional muscle mass independent of lipid and fibrotic tissue, both of which increase with advancing age^41^ and reduce the accuracy of the measurement of purely anatomic muscle mass using radiographic imaging or using indirect methods like DXA.

Newly published data from the ongoing MrOS study^42^ demonstrate that in older men, those with the lowest muscle mass/weight by D_3_‐Cr dilution have the highest risk of incident mobility limitation and injurious falls, and much worse physical performance and lower strength than those with higher muscle mass. These associations were not explained by confounding factors such as age, co‐morbidities, or activity level. In the same study, DXA based measures of lean mass had much weaker (if any) associations with these outcomes. This is the first large cohort study with a side by side comparison of DXA estimates and D_3_‐Cr muscle mass measurements on outcomes related to functional capacity and disability. These powerful associations between D_3_‐Cr dilution estimate of muscle mass and functional capacity and disability provides evidence that D_3_‐Cr‐muscle mass is an assessment of functional muscle mass that is not available using DXA estimates of whole body lean mass or appendicular lean mass. In an accompanying editorial, Schaap^43^ wrote, ‘In contrast to DXA, deuterated creatine (D3‐creatine) assesses muscle mass directly’ and ‘By isolating contractile muscle mass from noncontractile components including fat, the D3‐creatine assessment is not only an accurate method to assess muscle mass but is less biased by obesity and aging than DXA ALM’.

A particular value of D_3_‐creatine dilution is that it can be assessed in large number of subjects with very little subject burden. A subject simply swallows a capsule and produces a fasting urine sample later.^36^, ^42^ This ease of use may allow the D_3_‐creatine dilution method to be used in large subject and patient populations including many where CT or MRI cannot be used, such as paediatric or international studies in malnourished populations. For longitudinal trials, there is no limit to how many repeat measurements can be made as long as a urine sample is collected prior to subsequent doses of D_3_‐creatine to correct for baseline enrichment of urine D_3_‐creatinine.

## The ‘streetlight effect’ in muscle measurement

The initial definition of sarcopenia^44^ was an age‐related decrease in skeletal muscle mass, assuming that this loss, similar to the age‐related decrease in bone density and its association with fracture risk, would be strongly associated with late‐life dysfunction. While there is no disagreement that the loss of skeletal muscle mass occurs with advancing age, the lack of an accurate measurement of muscle mass has likely resulted in an underestimate of the effects of skeletal muscle mass on risk of late life disability and chronic disease. The term ‘streetlight effect’ comes from the old joke about the intoxicated man who is asked why he is searching for his lost wallet under the streetlight, rather than where he thinks he dropped it. The answer is, ‘Because the light is better here’. This describes the observational bias when investigators only search for something where it is easiest to look.^45^ Because there had been no method to accurately measure skeletal muscle mass in large clinical trials, DXA lean mass has been used as a surrogate measure of muscle mass. The use of the easily available DXA estimate of lean mass, and the assumption that the measurement of LBM is the same as measuring muscle mass, represents a *streetlight effect*. It has led to inconsistent results and potentially erroneous conclusions about the importance of skeletal muscle mass in health and disease. In contrast, the D_3_‐Cr dilution method provides an accurate, non‐invasive, and easy‐to‐use assessment of functional muscle mass. Continued use of this method should be considered in longitudinal observation and randomized controlled trials to better understand the role of changes in muscle mass in the aetiology of age‐associated loss of functional capacity and development of chronic diseases. The potential of the continued use of this method in prospective cohort studies is that a single diagnostic criterion of sarcopenia as skeletal muscle mass corrected for body mass may emerge as a powerful risk factor for late life disability and chronic disease.

## Conflict of interest

No conflicts are reported for the authors. Drs Hellerstein and Evans are listed as co‐inventors on the filed patents for the D_3_‐creatine dilution method; however, they do not derive any income related to commercial use of this method nor do they control the intellectual property. The authors certify that they comply with the ethical guidelines for authorship and publishing of the Journal of Cachexia, Sarcopenia and Muscle.^46^
